# Chemoprevention of prostate cancer in men with high-grade prostatic intraepithelial neoplasia (HGPIN): a systematic review and adjusted indirect treatment comparison

**DOI:** 10.18632/oncotarget.16230

**Published:** 2017-03-15

**Authors:** Kang Cui, Xiangnan Li, Yabing Du, Xiance Tang, Seiji Arai, Yiwei Geng, Ying Xi, Han Xu, Yue Zhou, Wang Ma, Tengfei Zhang

**Affiliations:** ^1^ Department of Oncology, The First Affiliated Hospital of Zhengzhou University, Zhengzhou, Henan, China; ^2^ Department of Thoracic Surgery, The First Affiliated Hospital of Zhengzhou University, Zhengzhou, Henan, China; ^3^ Department of Medical Affairs, Henan Cancer Hospital, Affiliated Cancer Hospital of Zhengzhou University, Zhengzhou, Henan, China; ^4^ Department of Hematology and Oncology, Beth Israel Deaconess Medical Center, Harvard Medical School, Boston, Massachusetts, United States; ^5^ Department of Urology, Gunma University Graduate School of Medicine, Maebashi, Japan; ^6^ Department of Breast Surgery, The First Affiliated Hospital of Zhengzhou University, Zhengzhou, Henan, China; ^7^ Deparmtent of B-Ultrasound, The First Affiliated Hospital of Zhengzhou University, Zhengzhou, Henan, China

**Keywords:** HGPIN, prostate cancer, chemoprevention, adjusted indirect meta-analysis, green tea catechins

## Abstract

**Background:**

High-grade prostatic intraepithelial neoplasia (HGPIN) is the precursor or premalignant form of prostate cancer. At least 30% patients with a confirmed HGPIN will develop prostate cancer within 1 year after repeated biopsy. HGPIN patients are the appropriate at-risk population for chemoprevention strategies investigation against prostate cancer. However the commonly used chemoprevention agents that targeted on hormonal imbalance or lifestyle-related factors showed varied results in HGPIN patients.

**Methods:**

Literature searches were conducted in PubMed, EMBASE and Cochrane library according to Cochrane guidelines before January 31^st^, 2017. Direct meta-analysis were performed to summarize the efficacy of candidate chemopreventative agents Dutasteride, Flutamide, Toremifene, Selenium, Green tea components, Lycopene and natural food products combination. Adjusted indirect meta-analyses were employed to compare the relative efficacy of these candidate chemoprevention agents head-to-head.

**Results:**

The overall incidence of prostate cancer in HGPIN was slightly decreased by chemoprevention agents (25.7% vs 31.5%, RR = 0.92, 95% CI: 0.83-1.03, P = 0.183), with minor heterogeneity (I^2^ = 22.3%, χ^2^ = 15.08, *P* = 0.237), but without statistical significance. Subgroup analysis showed that green tea catechins significantly decreased prostate cancer in HGPIN patients (7.60% vs 23.1%, RR = 0.39, 95% CI: 0.16-10.97, *P* P = 0.044), with moderate heterogeneity (I^2^ = 47.9%, χ^2^ = 1.92, *P* = 0.166). The adjusted indirect meta-analysis favored green tea catechins over other chemoprevention agents, and significantly when compared to natural food products combination (RR = 0.355, 95% CI: 0.134-0.934).

**Conclusion:**

The overall efficacy of chemoprevention agents in HGPIN patients is limited. But Green tea catechins showed the superiority to decrease prostate cancer in HGPIN patients.

## INTRODUCTION

Prostate cancer is the second most common cancer and the fifth leading cause of cancer-related death in men worldwide [1]. It is urgent to develop effective chemoprevention agents that are expected to decrease prostate cancer risk, delay or prevent surgery or chemo-radiotherapy, improve quality of life and decrease the frequency of invasive surveillance procedures [2, 3]. High-grade prostatic intraepithelial neoplasia (HGPIN) is considered as the precursor or premalignant form of prostate cancer with cytological changes similar to those in invasive prostate [2, 3]. HGPIN is diagnosed with atypical secretory luminal cells present by preexisting ducts and acini [4]. At least 30% patients with a confirmed HGPIN will develop prostate cancer within 1 year [5, 6]. Therefore, HGPIN patients are the appropriate at-risk population for chemoprevention strategies investigation.

The prostate cancer carcinogenesis is a complex process driven by genetic and epigenetic alterations [7, 8]. Hormonal imbalance and lifestyle-related factors are considered as the major contributors to prostate cancer [5, 9]. Regarding to hormonal imbalance, 5α-dihydrotestosterone (DHT), androgen receptor (AR), female hormones estrogens are critical targets for prostate cancer chemopreventions [9–11]. 5-α-reductase catalyzes the production of DHT. Then with increased androgenic activity, DHT activates multiple genes involved in carcinogenesis via AR [9–11]. Estrogen promoted the prostate cancer development by mediating estrogen receptors (ER) [12]. 5-α-reductase inhibitor Dutasteride, Finasteride, AR antagonist Flutamide, Bicalutamide and ER blocker Toremifene are investigated as chemoprevention agents for prostate cancer in clinical trials [2, 3, 13–17]. Due to the anti-oxidant and anti-proliferation activity and the limited toxicities, natural food compounds Selenium, Vitamin E, soy diets, tomato and green tea have been considered as the ideal candidate chemoprevention agents for prostate cancer in clinical trials [8, 18–23].

However, clinical trials performed with these agents in patients with HGPIN showed varied results. Most of these agents only showed minor effects to prevent prostate cancer development in HGPIN patients, for instance the Selenium and Vitamin E Cancer Prevention Trial (SELECT) [24, 25]. Even higher prostate cancer prevalence was found in HGPIN patients after taking some agents, for example Flutamid and the combination of lycopene, selenium, and green tea catechins [14, 26]. Moreover, it's important to find the agents with satisfying preventative efficiency to improve the clinical benefits for HGPIN patients. But due to the absence of head-to-head trials, network meta-analysis using direct and indirect evidence to compare the relative efficacy of these candidate agents is needed. In this study, We aimed to evaluate these chemoprevention agents and find the agents with the most satisfying efficiency.we performed a pairwise meta-analysis to summarize the efficacy of candidate chemoprevention agent, and used adjusted indirect meta-analysis to compare the relative efficacy of these candidate chemoprevention agents. We aimed to systematically summarize the efficacy of pharmacological agents and natural food compounds for HGPIN patients and find the agents with the most satisfying efficiency.

## RESULTS

### Characteristics of included trials

**Figure 1:**
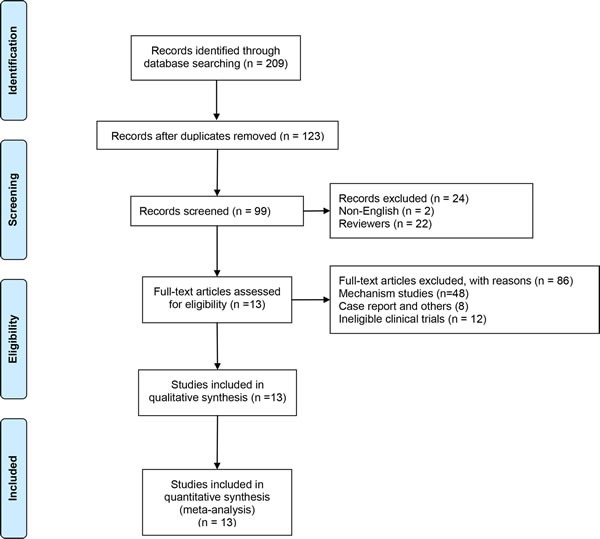
Flow diagram of study selection process

After removing duplicated literatures, unrelated literatures and some ineligible literatures, two investigators identified articles eligible for further review by screening titles and abstracts independently. Finally, we identified 13 literatures involved 3,020 patients eligible for analysis (Figure 1). Candidate chemoprevention agents used in these eligible studies included 5-α reductase inhibitor Dutasteride (*n* = 1), AR antagonist Flutamide (*n* = 1), Bicalutamide (*n* = 2), estrogen receptors blocker Toremifene (*n* = 2), micronutrient supplement Selenium (*n* = 1), green tea catechins (*n* = 2), Lycopene (*n* = 2), the natural food product combination (*n* = 2, Lycopene + Selenium + green tea catechins and soy protein + Vitamin E + Selenium). The details about the thirteen literatures are listed in Table 1.

**Table 1:** Summary of clinical trials involved in the meta-analysis

Authors	Year	Chemoprevent Reagent	Dose	Time	Patient	Race	Prostate cancer		Ref
							Treated	Control	
Milonas	2016	Dutasteride	50 mg/day	36	HGPIN	Caucasian	39/68	52/83	13
Kumar	2015	Green tea catechins	EGCG 400 mg/day	12	HGPIN/ ASAP	White & African American	5/49	9/48	8
Gontero	2015	Natural food product combination	lycopene 35 mg+ selenium 55 mg+ GTCs 600 mg/day	6	HGPIN/ASAP	--	10/27	3/26	25
Gann	2015	Lycopene	30 mg/day	6	HGPIN	White & African American	4/26	4/32	20
Taneja	2013	Toremifene	20mg/day	36	HGPIN /ASAP	White & African American	229/709	249/717	15
Marshall	2011	Selenium	200 mg/day	36	HGPIN	White & African American	48/135	49/134	25
Fleshner	2011	Natural food product combination	Soy protein 40g + vitamin E 800U + selenium 200g/day	36	HGPIN	--	41/156	39/147	24
Zanardi	2009	Bicalutamide	50 or 100mg/week	6	HGPIN	--	1/8	3/6	16
Bono	2007	Bicalutamide	50 mg/day	6	HGPIN	--	1/20	1/22	17
Mohanty	2007	Lycopene	8 mg/day	12	HGPIN	--	2/20	6/20	21
Price	2006	Toremifene	20-60mg/day	12	HGPIN	White & African American	31/109	15/308	3
Bettuzzi	2006	Green tea catechins	600mg/day	12	HGPIN	White & African American	1/30	9/30	5
Alberts	2006	Flutamide	250 mg/day	12	HGPIN	--	4/30	3/30	14

**Table 2:** Indirect comparison of chemoprevention agents under fixed effects

	Dutasteride	Green tea catechins	Natural food combination	Lycopene	Toremifene	Selenium	Flutamide	Bicalutamide
Dutasteride	1	2.33 (0.91, 6.00)	0.83 (0.53, 1.29)	1.31 (0.48, 3.57)	1.00 (0.74, 1.36)	0.94 (0.50, 1.76)	0.69 (0.16, 2.88)	0.06 (0.42, 10.1)
Green tea catechins	0.43 (0.17, 1.10)	1	0.35 (0.13, 0.94)*	0.65 (0.23, 1.86)	0.43 (0.17, 1.08)	0.40 (0.14, 1.18)	0.30 (0.06, 1.57)	0.88 (0.15, 5.39)
Natural food combination	1.21 (0.77, 1.88)	2.82 (1.06, 0.47)*	1	1.47 (0.52, 4,12)	1.21 (0.83, 1.78)	1.14 (0.58, 2.23)	0.83 (0.19, 3.55)	2.32 (0.47 11.5)
Lycopene	0.77 (0.28, 2.09)	1.78 (0.47, 6.73)	0.63 (0.23, 1.78)	1	0.77 (0.29, 2.05)	0.72 (0.23, 2.22)	0.53 (0.10, 3.55)	1.58 (0.25, 9.92)
Toremifene	1.00 (0.74, 1.34)	2.32 (0.93, 5.82)	0.83 (0.56, 1.21)	1.30 (0.49, 3.47)	1	0.94 (0.52, 1.69)	0.68 (0.17, 2.82)	2.05 (0.41, 11.6)
Selenium	1.06 (0.57, 1.99)	2.48 (0.85, 7.23)	0.88 (0.45, 1.72)	1.39 (0.45, 4.27)	1.07 (0.59, 1.92)	1	0.73 (0.16, 3.33)	2.19 (0.41, 11.6)
Flutamide	1.46 (0.35, 6.11)	3.40 (0.64, 18.1)	1.21 (0.28, 5.16)	1.90 (0.34, 10.5)	1.43 (0.35, 5.91)	1.37 (0.30, 6.27)	1	2.99 (0.37, 24.6)
Bicalutamide	0.49 (0.10, 2.37)	1.13 (0.19, 6.91)	0.40 (0.08, 2.00)	0.64 (0.10, 4.00)	0.49 (0.10, 2.35)	0.46 (0.09, 2.42)	0.33 (0.04, 2.74)	1

### Risk of bias

**Figure 2:**
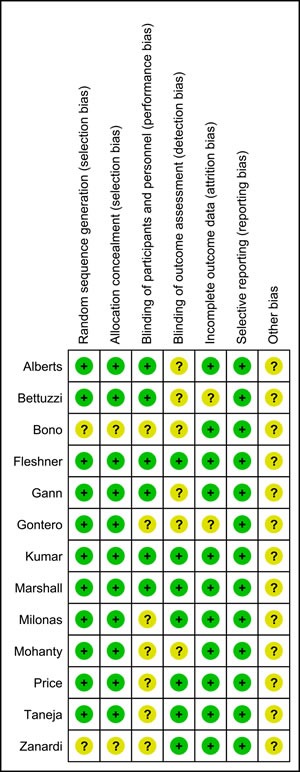
Risk of bias evaluations

No high risk of bias was assessed in these studies. The risk of bias evaluations for the included studies was summarized in Figure 2.

### Direct meta-analysis of the efficacy of chemoprevention agents for HGPIN

**Figure 3:**
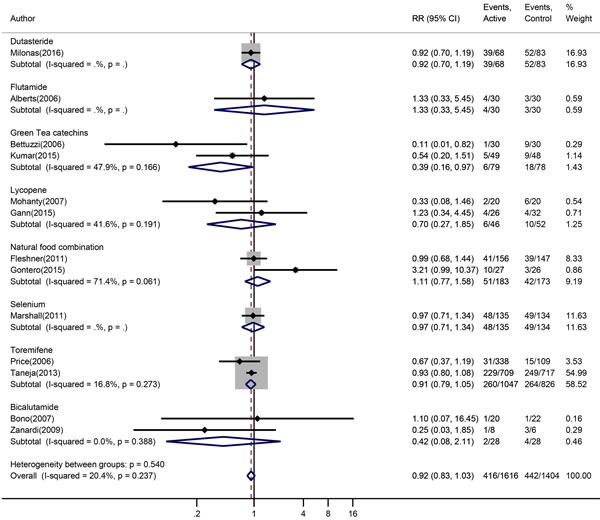
Forest plot for risk ratio confidence intervals of prostate cancer incidence in HGPIN patients The red squares indicated the single risk ratios reported from each study. The black diamond squares indicated the pooled risk ratios from meta-analysis.

**Figure 4:**
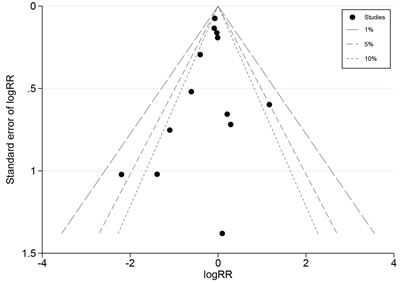
Funnel plot

The pooled prostate cancer incidence in HGPIN patients received chemoprevention agents and patients not received chemoprevention agents were 25.7% and 31.5% respectively. The overall incidence of prostate cancer in HGPIN was slightly decreased by chemoprevention agents (RR = 0.92, 95% CI: 0.83-1.03, *P* = 0.183), with minor heterogeneity (I^2^ = 22.3%, *x*^2^ = 15.08, *P* = 0.237), but without statistic significance (Figure 3). Begg's test and Egger's test both showed no evidence of substantial publication bias (*P* = 0.721, *P* = 0.788). The funnel plot was shown in Figure 4.

Subgroup analysis showed that green tea catechins significantly decreased the prostate cancer in HGPIN patients (7.60% vs 23.1%, RR = 0.39, 95% CI: 0.16-10.97, *P* = 0.044), with moderate heterogeneity (I^2^ = 47.9%, *x*^2^ = 1.92, *P* = 0.166). Lycopene, Toremifene, Bicalutamide decreased the prostate cancer in HGPIN patients (13.0% vs 19.2% RR = 0.70, 95% CI: 0.27-1.85, 24.8% vs 32.0% RR = 0.91, 95% CI: 0.79-1.05, 7.2% vs 14.3% RR = 0.42, 95% CI: 0.08-2.11 respectively), but without statistical significances. The natural food combination showed slight increased prostate cancer in HGPIN patients (7.60% vs 23.1%, RR = 1.11, 95% CI: 0.77-1.58) without statistical significance.

### Adjusted indirect meta-analysis of the efficacy of chemoprevention agents for HGPIN

**Figure 5:**
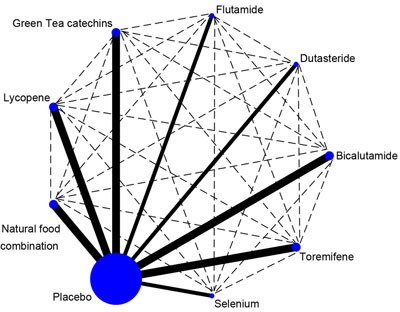
The network in the adjusted indirect analysis The full line indicates the direct analysis and the dotted line indicates the indirect analysis.

The network is given in Figure 5. We compared each pair of chemoprevention agents using placebo as a data bridge head-to-head. The indirect estimates favor green tea catechins over other chemoprevention agents, and significantly when compared to natural food products combination (RR = 0.355, 95% CI: 0.134, 0.934). Lycopene showed better chemoprevention effects in HGPIN patients than Dutasteride, natural food combination, Toremifene, Selenium and Flutamide, but without statistical significance. Regarding to the chemoprevention agents targeted on hormonal imbalance, the indirect estimates favor Bicalutamide over Dutasteride, Toremifene and Flutamide, but without statistical significance. Toremifene also showed better chemoprevention effects than Flutamide, but without statistical significance. All heterogeneity statistics are not significant at *P* = 0.05.

## DISCUSSION

In this systematic review, we combined direct evidence from 13 randomized controlled trials comparing 7 different interventions and reporting on 3,020 participants with HGPIN to make observations regarding the potential efficacy of prostate cancer chemoprevention agents. The meta-analysis showed that the efficacy of prostate cancer chemoprevention agents is not satisfying, it only slightly decreased the prostate cancer and without significance. However, compared to other agents, green tea catechins showed promising benefits for HGPIN patients, and significantly better than the natural food product combination.

Our study extends findings from primary randomized controlled trials and systematic reviews. Most of the study for prostate cancer prevention focused on the observation or retrospective study to investigate the risk factors for prostate cancer, or the effects of candidate agents to prevent prostate cancer progression, recurrence and mortality. Chemoprevention agents targeted on pre-cancer patients to decrease prostate cancer risk is also important to improve the health outcomes. An ideal preventive agent should delay or reverse carcinogenesis effectively, at the same time, it should not cause any harm to healthy people, which raised the bar for chemoprevention research. Former studies showed that some 5-α reductase inhibitor showed significant effects to reduce the risk of prostate cancer, but with increased risk for high-grade disease [27–29]. For another, HGPIN patients are at high risk to develop prostate cancer [5, 6], but these studies are performed on general risk population but not HGPIN patients. Clinical trials to investigate these agents and develop novel chemoprevention agents in HGPIN population were needed. In this study, we summarized the findings from primary randomized controlled trials to get a more clear data about chemoprevention of prostate cancer in HGPIN patients.

The chemoprevention agents summarized in this study are targeted on hormonal imbalance or diets, the two important contributors to prostate cancer [5, 9]. Agents targeted on hormonal imbalance summarized in this study include Dutasteride [13], Flutamide [14], Bicalutamide [16, 17] and Toremifene [3, 15]. The Dutasteride clinical trial involved in this study showed that Dutasteride did not decrease prostate cancer incidence but did not worsen the HGPIN [13]. In the Flutamide trial, no evidence of benefit was found [14]. The pooled results from meta-analysis showed the potential of Bicalutamide to inhibit prostate cancer development, but without significance [16, 17]. The pooled efficacy of Toremifene was also limited in HGPIN patients [3, 15]. However, the adjusted indirect analysis showed that Bicalutamide might have better clinical benefits for HGPIN patients than Dutasteride, Toremifene and Flutamide. This result might be related to the different mechanisms among these drugs. Bicalutamide and Flutamide are AR antagonists. AR plays a pivotal role in prostate cancer by transactivation of multiple genes involved in tumorigenesis [30]. Moreover, Bicalutamide was derived from flutamide by structural modification to increase the agonist properties against AR [31]. However, the limitation of these chemical drugs underscored the needs of other chemopreventive agents for HGPIN patients.

Natural products such as vitamins, minerals, probiotics, herbal medicines showed potential cancer prevention effects with low toxicity profiles [32, 33]. “Food-based” prevention approaches such as green tea, soy product, lycopene, selenium included in this meta-analysis are all reported to be associated with reduced risk of cancer. In our direct meta-analysis, subgroup analysis showed that only green tea catechins showed significant clinical benefits for HGPIN patients. In our indirect meta-analysis, the results favor green tea catechins over all other chemoprevention agents. Observation studies in Japan and China showed that green tea intake in diet might decrease the risk of localized and advanced prostate cancers [34–36]. But some studies showed no association between tea consumption and prostate cancer risk [37, 38]. A recent meta-analysis did not support the conclusion that tea consumption could reduce prostate cancer risk neither [39]. The data used in this meta-analysis were extracted from cohort studies and case-control studies, but not randomized controlled trials. When they limited the analysis to case-control study, they found a protective effect for tea consumption against prostate cancer. Our results pooled results from randomized controlled trials supported the conclusion that green tea catechins was an efficient agent for HGPIN patients to prevent prostate cancer. (−)-epigallocatechin-3-gallate (EGCG) are the most abundant green tea catechins, comprising 50% to 75% of the material in the investigational agent in the two trials [5, 8]. EGCG showed anticancer activity for prostate cancer in cell model, animals and human clinical trials [5, 8, 40]. EGCG can decreased androgen receptor (AR) and inhibit 5-a-reductase. It also showed the effects to inhibit proliferation, angiogenesis, apoptotic cell death and other cell activity through oncogenic signaling pathway such as NFκB/MAPK/IGFR/COX-2 [40]. Since carcinogenesis is a process with multiple stages and multiple factors involved, the synergistic effects of EGCG may contribute to the superior benefits of EGCG than other chemoprevention agents.

Our adjusted indirect meta-analysis showed that lycopene might have better chemoprevention effects in HGPIN patients than most of the other agents except green tea catechins and Bicalutamide, but without significance. Lycopene has been studies for a long time because of their efficacy to lower the risk of prostate cancer [41]. Between the two lycopene trials in this meta-analysis, the HGPIN patients were given the different dose, at 8 mg/day in Mohanty's study and 30mg/day in Gann's study [20, 21]. But the lower dose showed the better clinical benefit. In the two natural production combination trails, each single components all have the potential to prevent prostate cancer, but the combination even worsen the prostate cancer incidence [24, 26]. The failure of high dose lycopene and the combination in Gontere's study may due to the short time intervention, only 6 months. We performed a subgroup Meta-analysis to compare trials with 6 months intervention and that with more than 6 month, and found no significant difference between them (Supplemental Figure 1). But this subgroup setting is not thorough due to the limited biological significance. Recent data showed that a high prostate cancer detection rate at early repeat biopsy (6 months) in men with HGPIN is common in several clinical trials, because prostate cancer has been already present at the initial HGPIN diagnosis [13]. This may help to explain the reason why 6 month intervention showed limited chemoprevention effects. Larger, longer and précised design trials are needed to figure out the reason.

There are limitations in this study. A recent study to systematically summarize dietary, nutritional, and physical activity interventions for the prevention of prostate cancer progression and mortality failed to get a conclusion. The author considered limitation patients numbers, risk of bias, underpowered, inadequately reported, short duration or measured surrogate outcomes of unproven relationship to mortality or disease progression as the potential reasons [42]. These issues may be common in modification interventions. Although we didn't find significant risk bias in the analysis, the above reasons may exist. HGPIN may be more prevalent in African American men even after controlling other factors [43]. However most of the patients recruited in these trials are white men. Prostate cancer incidence was significantly higher in plurifocal HGPIN patients than monofocal HGPIN patients [44]. The number of core samples with HGPIN increase is in parallel with the risk for prostate cancer [45]. Over diagnosis of HGPIN influenced the results from the clinical trials taking candidate agents to prevent development of prostate cancer [46]. However in this meta-analysis, we didn't analysis the race, histopathology status, the number of core samples and the over diagnosis issues about HGPIN, which are potential to cause the bias of the results. Due to the limited data, we only assess the prostate cancer incidence but not other indicators such as the disease free survival, the PSA changes, and adverse effects as additional endpoint.

In conclusion, this study didn't show a significant effect of the chemoprevention agents to prevent prostate cancer development in HGPIN patients. The direct and indirect meta-analysis demonstrated that green tea green tea catechins significantly inhibit prostate cancer in HGPIN patients and are better than other chemoprevention agents for prostate cancer. The results of this study have implication for the prostate cancer prevention and support the future clinical study with green tea catechins for HGPIN patients.

## MATERIALS AND METHODS

### Search strategy and searches

Literature searches were conducted in PubMed, EMBASE and Cochrane library according to Cochrane guidelines before January 31^st^, 2017 [47]. Search key terms included high-grade prostatic intraepithelial neoplasia, chemoprevention, prostate cancer and randomized controlled trial. We also manually searched references in identified studies in case of missing trials.

### Study selection

Randomized controlled trials that enrolled high-grade prostatic intraepithelial neoplasia patients to receive any intervention for prostate cancer chemoprevention were included. The primary outcome assessed in this study is the prevalence of prostate cancer in high-grade prostatic intraepithelial neoplasia patient after chemoprevention intervention. The eligible studies should report the incidence of prostate cancer development after the intervention. The risk of bias was assessed by three reviewers using the Cochrane Collaboration's tool [48]. The risk of bias was graded as high, low, or unclear.

### Statistical method

Direct meta-analysis was performed with fixed effects model to estimate pooled relative ratios and 95% confidence intervals incorporating heterogeneity within and between studies. Subgroup analysis was based on the different chemoprevention agents. Statistical heterogeneity was assessed with I^2^ statistic, with values over 50% indicating substantial heterogeneity. Publication bias was analyzed by both Begg's and Egger's regression asymmetry test, and visually evaluated using the funnel plot.

Adjusted indirect comparisons were performed to estimate the relative ratio between different candidate chemoprevention agents using placebo as a data bridge. The adjusted indirect meta-analysis developed by Bucher et al. allows for the comparison of two treatments by comparing each of the interventions with a common comparator [49]. The “indirect” command implemented in Stata was employed to convert the summary estimates (log RRs) and measures of uncertainty (variances) from the meta-analyses into an RR and 95% CI representing the difference between each two chemoprevention agents [50]. Stata Statistical Software (version 14.0 Stata Corp., College Station, TX, USA) was used for all analyses. A two-sided P value ≤ 0.05 was considered as statistically significant. The data set and command used in Stata are list in Supplemental Table 1.

## SUPPLEMENTARY TABLE AND FIGURE



## Abbreviations

HGPIN: High-Grade Prostatic Intraepithelial Neoplasia
RR: risk ratio
CI: confidence interval
DHT: 5α-dihydrotestosterone
AR: androgen receptor
PSA: Prostate-specific antigen
