# Supplementary material for: Chemoprevention of prostate cancer in men with high-grade prostatic intraepithelial neoplasia (HGPIN): a systematic review and adjusted indirect treatment comparison
Source: Oncotarget. 2017 Mar 15;8(22):36674–84. doi: 10.18632/oncotarget.16230 (PMC5482687; doi:10.18632/oncotarget.16230)
Supplement: Supplementary file 1 [file oncotarget-08-36674-s001.pdf]

Chemoprevention of prostate cancer in men with high-grade prostatic intraepithelial neoplasia (HGPIN): a systematic review and adjusted indirect treatment comparison

Supplementary Materials

Supplementary Table 1: Data sheet for analysis and the commons used in Stata

study	year	t1[a]	t2[b]	pcatr [c]	treatment [d]	pcactrl [e]	control [f]	time	subgroup2 [g]	order [h]
Milonas	2016	Dutasteride	Placebo	39	29	52	31	36	2	0
Gann	2015	Lycopene	Placebo	4	22	4	28	6	1	1
Gontero	2015	Natural food combination	Placebo	10	17	3	23	6	1	1
Kumar	2015	Green Tea catechins	Placebo	5	44	9	39	12	2	1
Taneja	2013	Toremifene	Placebo	229	480	249	468	36	2	1
Fleshner	2011	Natural food combination	Placebo	41	115	39	108	36	2	1
Marshall	2011	Selenium	Placebo	48	87	49	85	36	2	1
Zanardi	2009	Bicalutamide	Placebo	1	7	3	3	6	1	1
Bono	2007	Bicalutamide	Placebo	1	19	1	21	6	1	1
Mohanty	2007	Lycopene	Placebo	2	18	6	14	12	2	1
Alberts	2006	Flutamide	Placebo	4	26	3	27	12	2	1
Bettuzzi	2006	Green Tea catechins	Placebo	1	29	9	21	12	2	0
Price	2006	Toremifene	Placebo	31	307	15	94	12	2	1

Table 2	indirect logRR SE t1 t2, fixed eff(RR) eform trta(t1) trtb(t2)
Figure 3	metan pcatr treatment pcactrl control, rr fixedi lcols (study) sortby (year)counts group1 (Active) group2 (Control) textsize(100) boxsca(80) xlab(0.2,1,4, 8,16) graphregion(color(white)) plotregion(color(white)) by (t1)
Figure 4	metafunnel logrr [i] _selogES [j], xtitle(logRR) ytitle(Standard error of logRR)
Figure 5	networkplot t1 t2, noweight
Supplemental Figure 1	metan pcatr treatment pcactrl control, rr fixedi lcols (study) sortby (year)counts group1 (Active) group2 (Control) textsize(100) boxsca(80) xlab(0.2,1,4, 8,16) graphregion(color(white)) plotregion(color(white)) by (subgroup2)

[a] the chemoprevention agents used in clinical trial; [b] the control group; [c] the number of patients developed prostate cancer in chemoprevention agents intervention group; [d] the number of patients without prostate cancer in chemoprevention agents intervention group; [e] the number of patients developed prostate cancer in control group; [f] the number of patients without prostate cancer in control group; [g] the intervention time; [f] 0=6 months, 1= > 6 months; [i,j] these two data will be generated by the commons list in Figure 3

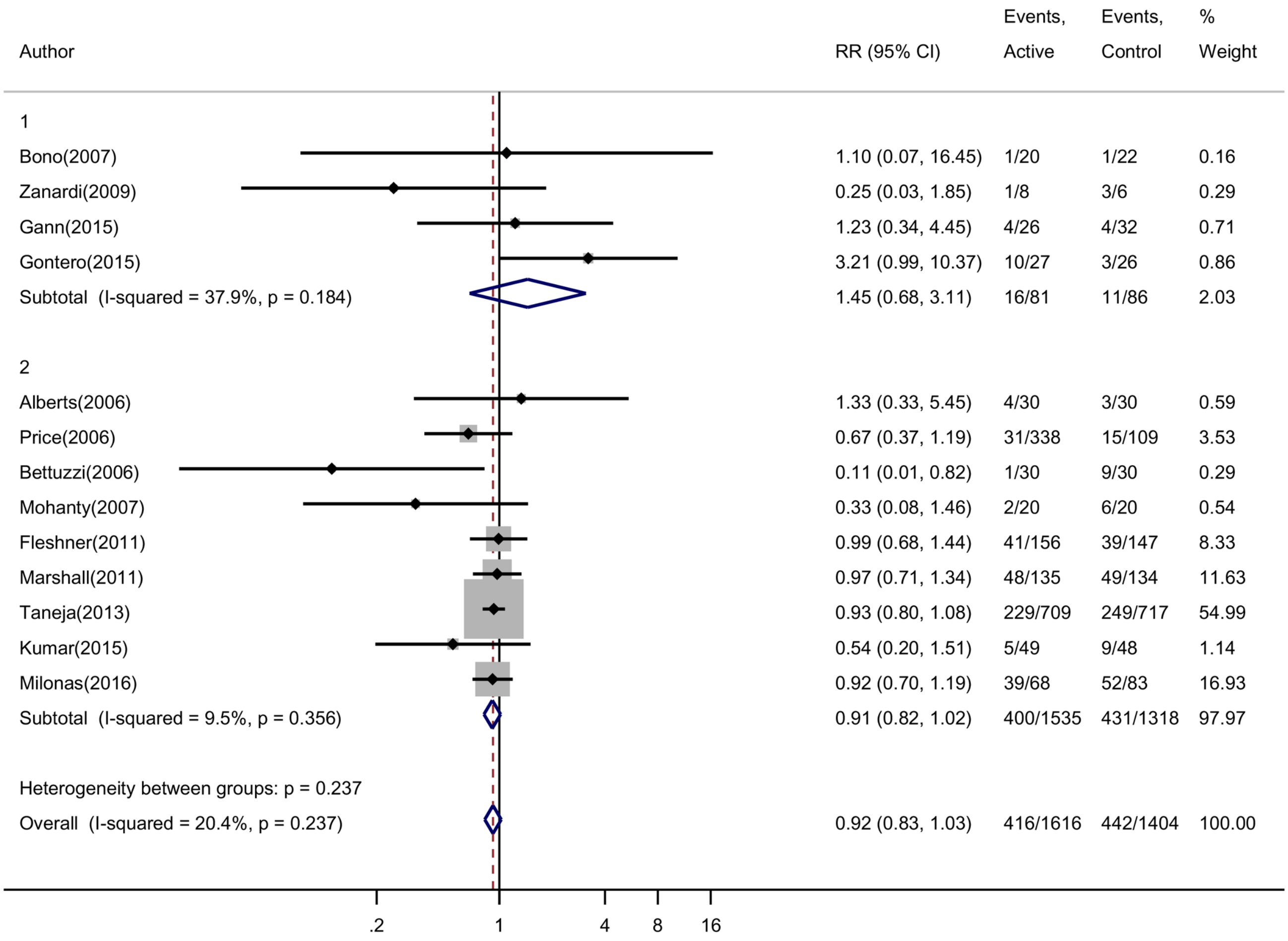

Supplementary Figure 1
